# Iron as a Therapeutic Target in *HFE*-Related Hemochromatosis: Usual and Novel Aspects

**DOI:** 10.3390/ph11040131

**Published:** 2018-11-26

**Authors:** Olivier Loréal, Thibault Cavey, François Robin, Moussa Kenawi, Pascal Guggenbuhl, Pierre Brissot

**Affiliations:** INSERM, Univ Rennes, INRA, CHU Rennes, Institut NUMECAN (Nutrition Metabolisms and Cancer), F-35033 Rennes, France; thibault.cavey@chu-rennes.fr (T.C.); francois.robin@chu-rennes.fr (F.R.); moussa.kenawi@univ-rennes1.fr (M.K.); pascal.guggenbuhl@univ-rennes1.fr (P.G.); pierre.brissot@univ-rennes1.fr (P.B.)

**Keywords:** iron, genetic hemochromatosis, non transferrin bound iron, hepcidin, ferroportin, venesections

## Abstract

Genetic hemochromatosis is an iron overload disease that is mainly related to the *C282Y* mutation in the *HFE* gene. This gene controls the expression of hepcidin, a peptide secreted in plasma by the liver and regulates systemic iron distribution. Homozygous *C282Y* mutation induces hepcidin deficiency, leading to increased circulating transferrin saturation, and ultimately, iron accumulation in organs such as the liver, pancreas, heart, and bone. Iron in excess may induce or favor the development of complications such as cirrhosis, liver cancer, diabetes, heart failure, hypogonadism, but also complaints such as asthenia and disabling arthritis. Iron depletive treatment mainly consists of venesections that permit the removal of iron contained in red blood cells and the subsequent mobilization of stored iron in order to synthesize hemoglobin for new erythrocytes. It is highly efficient in removing excess iron and preventing most of the complications associated with excess iron in the body. However, this treatment does not target the biological mechanisms involved in the iron metabolism disturbance. New treatments based on the increase of hepcidin levels, by using hepcidin mimetics or inducers, or inhibitors of the iron export activity of ferroportin protein that is the target of hepcidin, if devoid of significant secondary effects, should be useful to better control iron parameters and symptoms, such as arthritis.

Iron metabolism must be tightly controlled in order to avoid deleterious situations, including iron overload diseases, especially *HFE*-related hemochromatosis. In such conditions an iron depletive treatment is engaged in order to avoid the development of complications. However, iron depletive therapy targets only iron excess and alternative, possibly complementary, novel approaches are needed.

## 1. Normal Iron Metabolism

### 1.1. The Ferroportin/Hepcidin Duo Controls Systemic Iron Metabolism

Systemic iron metabolism is characterized by a continuous distribution of iron from plasma toward cells which require its presence for participating in biological functions, including oxygen transport and enzymatic processes [1]. Plasma iron must be continuously renewed. Plasma iron originates predominantly from macrophages that recycle iron from senescent erythrocytes during the erythrophagocytosis process. The erythropoietic cells contains 70% of total body iron. The macrophages provide schematically 19 mg out of the 20 mg of iron required from the plasma by cells every day. The second source of iron is digestive absorption. Iron is absorbed in two steps from the nutrients. The first step takes place at the apical pole of the enterocytes, non-heme and heme iron being transferred from nutrients to the enterocyte cytoplasm. The transfer of non-heme iron implicates DMT1 (divalent metal transporter 1) that takes up ferrous iron [2] after reduction by the ferrireductase dCytb [3]. Heme iron is taken up through a receptor that could be HCP1 (heme carrier protein1) (controversial) [4,5,6]. The second step of iron absorption is the transfer of iron from the cytoplasm toward the plasma through the basal membrane of the enterocyte. This process limits the iron entry into the body, and only 1–2 mg are transferred each day toward the plasma, whereas the remaining iron is transiently stored within the cytoplasm and will be lost during enterocyte desquamation. This selective process is the classical mucosal block [7]. Ferroportin is the iron export protein that allows iron egress from enterocytes (concerning both the non-heme and heme iron absorbed at the apical side) and macrophages toward the plasma [8,9,10]. Once transferred into the plasma, and after its oxidation by ferroxidase enzymes—ceruloplasmin [11] and/or hephaestin [12]—ferric iron is associated to transferrin, the protein that can link up to two iron atoms and delivers iron to cells. Cellular uptake of transferrin iron is mediated by the transferrin receptor 1 (TFR1) [13]. Thereafter, endocytosis of the complex permits the transport of iron into endocytic vesicles and subsequently, its transfer to the cytosol by DMT1, that is also expressed in endocytic vesicles, after oxidation of iron by STEAP3 (*Six-Transmembrane Epithelial Antigen of the Prostate 3*) [14]. Another transferrin receptor, transferrin receptor 2 (TFR2) is expressed by cells, especially hepatocytes, but its affinity coefficient for transferrin is very low [15] so that its function is related to iron sensing rather than iron transport [16].

Hepcidin is a small cysteine rich peptide [17,18,19], mainly expressed by hepatocytes and secreted in the plasma, that interacts with ferroportin [20], the only known protein involved in cellular iron export. An increase of plasma hepcidin level leads to a decrease of both digestive iron absorption and iron leakage by macrophages [21]. Hepcidin decreases plasma iron concentration and the saturation of transferrin by iron. Conversely, a decrease of hepcidin expression favors cell membrane expression of ferroportin, and, in turn, the iron release from cells into the plasma, thus, increasing the transferrin saturation by iron. The regulation of hepcidin expression plays a major role in the maintenance of iron homeostasis.

### 1.2. Regulation of Hepcidin Expression

Hepcidin expression is regulated by many factors, including iron status [19] and inflammation [19,22] that induce an upregulation of hepcidin expression, and hypoxia/erythropoiesis activity [23,24,25] that decrease hepcidin expression. Mechanisms related to the induction of hepcidin expression by iron status are mainly transcriptional. 

**Figure 1:**
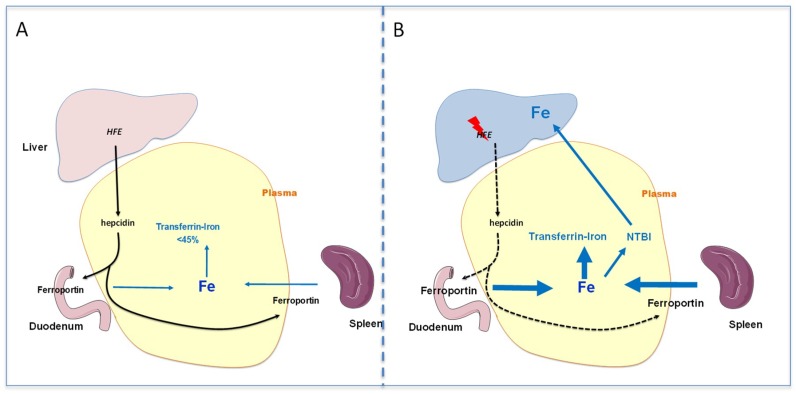
Schematic representation of the pathophysiological mechanisms leading to the development of iron overload during *HFE*-related hemochromatosis. (**A**): Normal situation with adequate *HFE* signaling allowing control of transferrin saturation level (<45%). (**B**): Genetic hemochromatosis with low activity of *HFE*-related signaling that limits hepcidin expression and in turn favors iron release in plasma, despite the presence of a sufficient amount of iron. Thus, the transferrin saturation increases and non-transferrin bound iron appears and targets organs such as liver, leading to abnormal iron accumulation.

The first iron-related mechanism that regulates hepcidin expression is reported to be linked to transferrin saturation level in plasma [26,27]. This pathway involves the *HFE* gene, located on the chromosome 6, that encodes a HLA like class I protein that is expressed on cell membrane in association with the ß2-microglobulin [28]. It has been reported that HFE protein may interact either with TFR1 [29,30] or TFR2. The mechanism potentially involved in such regulation is a decrease of the physical interaction between TFR1 and HFE proteins when transferrin saturation increases [31]. This could lead to the stimulation of a MAP (mitogen-activated protein kinase) signaling pathway that promotes the hepcidin transcription level [27]. However, while the HFE/TFR2 interaction has been documented in in vitro experiments, the in vivo relevance of these findings is questionable [32]. The increase of hepcidin expression promotes ferroportin degradation, and thus, reduces plasma iron concentration and transferrin saturation by iron (Figure 1). 

The second regulatory mechanism implicates the bone morphogenetic protein/hemojuvelin/son of mothers against decapentaplegic homolog (BMP/HJV/SMAD) pathway [33,34,35,36]. When cell iron concentration increases, BMP6 and BMP2 proteins are produced and secreted by hepatocytes and more likely sinusoidal cells [37,38,39]. BMPs interact with BMP receptor proteins that are associated to HJV, acting as BMPs co-receptor. The interaction induces a phosphorylation of SMADs (Son of Mothers Against Decapentaplegic) 1, 5, 8 that are translocated toward the nucleus after association with SMAD4 in the nucleus [40,41]. Then, they interact with BMP-responsive element sequence within the hepcidin gene promoter and promote hepcidin transcription [35,36,42]. 

It is noteworthy that the hemochromatosis proteins HFE and HJV form a membrane-associated protein complex for hepcidin regulation. This suggests that the two pathways are not fully independent [43,44], and further work should help to better specify the respective roles of the different actors.

## 2. Pathophysiology of *HFE* Hemochromatosis

### 2.1. HFE Hemochromatosis

*HFE* hemochromatosis is a disease mainly related to homozygosity of the *C282Y* (*p.Cys282Tyr*) mutation in the *HFE* gene [45]. The *p.Cys282Tyr* mutation alters the structure of the *HFE* protein due to the substitution of a cysteine that is engaged in intra-molecular disulfide bounds, that play a role in the protein shape, by a threonine. Thus, the expression of HFE protein on cell membrane, as well as its interaction with the Beta2 microglobulin are altered [29]. Some exceptional private mutations in the *HFE* gene can also lead to hemochromatosis, when present either at the homozygous state or in association with the *C282Y* mutation [46]. Homozygosity for *H63D* (*p.His63Asp*) that is present at homozygous state in approximately 2% of the Caucasian population cannot by itself generate clinically significant iron excess, so that, when associated to iron overload, one must search for associated genetic or acquired associated factors that promote iron excess [47]. It must be pointed out that the penetrance of *HFE*-related hemochromatosis is very incomplete. In terms of clinical penetrance, some studies have estimated a prevalence of 25–60%, while a single study reported a prevalence of 28% in males and 1% in females [48]. However, in the same study the biochemical penetrance, assessed by increased ferritinemia, was much higher (82% and 55%, respectively) [48].

### 2.2. Pathophysiology of Iron Overload during HFE Hemochromatosis

In hemochromatosis, iron overload is a two-hit phenomenon.

The first hit (Figure 1), which is related to the structural change of the protein is a deficiency in hepcidin expression and secretion—meaning hepcidin deficiency—compared to the iron stores [22,26]. In other words, hepcidin expression is lower than expected when considering plasma transferrin saturation and body iron stores [49]. This hepcidin deficiency results from the mutation in the *HFE* gene that alters the efficacy of the transduction pathway regulating hepcidin expression. Consecutively, despite iron excess, ferroportin expression on cell membranes of enterocytes and macrophages remains elevated and favors an increase of both plasma iron concentration and transferrin iron saturation [20]. As previously mentioned, transferrin iron ingress into cells is modulated by the expression level of its receptor TFR1. In physiological situation, TFR1 expression on cell membranes is downregulated when cellular iron is in excess [50], in order to avoid cellular iron accumulation with subsequent toxicity, especially through the production of reactive oxygen species (ROS) [51,52]. The iron responsive element/iron regulatory protein system (IRE/IRP) regulates TFR1 and ferritin expression, adapting iron entry into the cell (TFR1) and the capacity of iron storage in cells (ferritin), to the variations of cellular iron content [50,53]. 

The second hit (Figure 1) involved in the development of iron overload in hemochromatosis results from the appearance of the non-transferrin-bound form of iron (NTBI) [54]. Indeed, transferrin saturation increase favors the presence of NTBI in plasma [55]. The NTBI is constituted of low molecular forms of iron linked to citrate or acetate [56]. The NTBI, in contrast to transferrin iron, constantly enters the cells, especially through the Zip14 transporter [57], even when they are already overloaded [58,59], whereas transferrin iron ingress is physiologically reduced due to the decrease of TFR1 on cell membrane [60,61]. The transporters involved in the uptake of NTBI are mainly expressed in the liver, the pancreas [57,62], and the heart, explaining that these organs are the primary targets of iron excess.

It is important to note that rare or very rare non-*HFE* mutations may also favor hepcidin deficiency. Homozygous and compound heterozygous mutations in the *HAMP* [63] or *HJV* [64] genes induce an early and severe iron overload disease (juvenile hemochromatosis) that is related to severe hepcidin deficiency with major complications that quickly impact well-being and life expectancy. In addition, mutations in the *TFR2* gene induce an hepcidin deficiency that provokes a clinical iron overload phenotype which is in between juvenile hemochromatosis and the classical *HFE*-related hemochromatosis form [65].

### 2.3. Pathophysiology of Organ Damage in Hemochromatosis

Complications of *HFE*-related genetic hemochromatosis, and more globally of hemochromatosis related to hepcidin deficiency, include hepatic damage with the development of liver fibrosis, with the risks of cirrhosis and hepatocellular carcinoma, diabetes, and at a lesser degree, heart dysfunction, which are sources of morbidity and mortality [66,67]. The risk of hepatic fibrosis increases with the severity of iron overload [68], and it is recommended to perform a liver biopsy in patients exhibiting very high levels of ferritinemia [69]. This risk is associated with the presence of sideronecrotic lesions of hepatocytes [70], that likely corresponds to the recently identified ferroptotic cell death process [71,72], a new cell death pathway in cells containing high iron content. Whereas hepatocyte iron loading is the pathophysiological basic feature of iron overload in hemochromatosis, it is noteworthy that Kupffer cell iron load finally occurs in advanced iron hemochromatosis and also represents a risk for the development of hepatic damage [70]. The role of an iron-related induction of TGF (transforming growth factor)-beta in the development of fibrosis has been reported [73,74]. It should be underlined that hepatocellular carcinoma, that mostly develops in patients with a cirrhotic liver, can also be rarely found in non-cirrhotic patients which suggests the role of iron itself [75] and/or additional cofactors in the development of hepatocellular carcinoma (HCC) (see below). Finally, patients exhibiting cirrhosis before iron depletive treatment still present a risk for developing HCC even despite completion of iron depletive treatment [67]. 

Other complications may include hypogonadism—mostly in juvenile hemochromatosis—and much more frequently osteoporosis and arthritis that have a strong impact on the quality of life. Arthritis is characterized by absence of systemic inflammation and sometimes presence of calcium pyrophosphate crystals in the synovial fluids and visible on X-rays (review in [76]).

**Figure 2:**
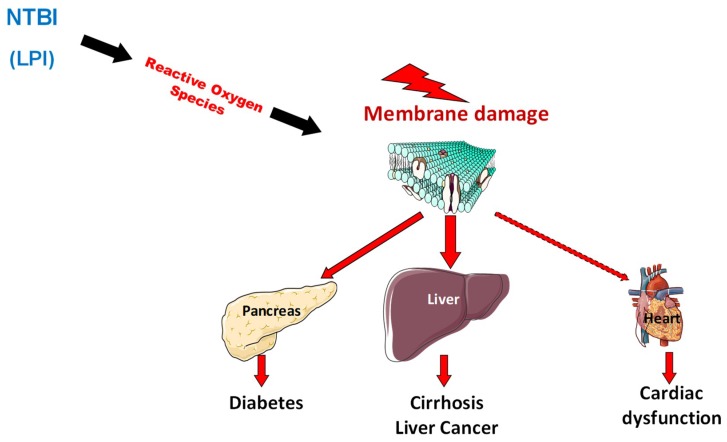
The appearance of non-transferrin bound forms of iron in plasma favors organ iron deposition but also the occurrence of oxidative stress that alters organelles in cells especially in iron overloaded organs.

The ability of excessive iron to generate oxygen reactive species (ROS) (Figure 2) through the Haber–Weiss and Fenton reactions is strongly involved in the development of tissue lesions [77]. Indeed, ROS induces peroxidation that alters lipids, proteins, and DNA, generating dysfunctions of organelles, including mitochondria and cells, thus leading to tissue and organ damage [78,79,80]. One of the abnormal forms of NTBI, called Labile Plasma iron-LPI (or reactive plasma iron), is found in plasma when transferrin saturation reaches 80% [81]. This iron species is highly reactive and participates strongly in oxidative stress. Labile plasma iron is considered as a major determinant in the development of organ damage during hemochromatosis [82].

In addition to the typical disease causing mutations associated with hemochromatosis, additional genetic modifiers have recently been described to elucidate in part, the large disparity in disease manifestations in patients. In this context, the importance of gene polymorphisms, e.g., GNPAT (glyceronephosphate O-acyltransferase) remains controversial and awaits additional confirmatory studies [83,84,85,86,87,88]. Alcohol consumption [68,89], non-alcoholic fatty liver disease [90], and viral hepatitis [91,92] may also be involved in disease penetrance, as well as a polymorphism in the *PCSK7* [93] or *PNPLA3* [94] genes.

## 3. Iron Is Presently the Main Therapeutic Target in *HFE*-Related Hemochromatosis

It is well known that iron depletion is a treatment of choice for hemochromatosis patients [95]. Indeed, when performed in patients prior to severe complications, the iron depletive treatment restores normal life expectancy, meaning that hepatic, pancreatic, and cardiac dysfunctions can be fully prevented [67]. In addition, partial regression of liver fibrosis after completion of iron removal has been reported [96].

### 3.1. Venesections Are the Mainstay Treatment for Iron Removal

**Figure 3:**
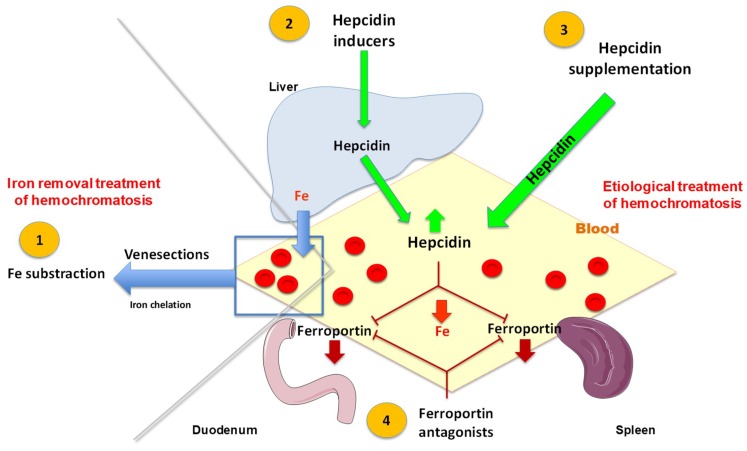
Schematic representation of currently used iron removal treatments during hemochromatosis and potential pathophysiological actions. (1) Iron depletive treatment is classically operated through repeated venesections that remove red blood cells. In some rare cases, iron chelators can be also used as complementary or suppletive treatment. (2) Hepcidin supplementation by endogenous or exogenous hepcidin. (3) Alternatively (4) the use of ferroportin antagonists could be also a way of treatment.

The principle of repeated venesections [47,95] (Figure 3) is to remove red blood cells that are known to be very rich in iron, as part of hemoglobin. Erythropoiesis is stimulated to compensate hematocrit loss, and iron is taken up from the plasma by erythroblasts to produce new erythrocytes. In order to maintain enough plasma iron for the stimulated erythropoiesis, iron is released from macrophages and enterocytes but also from iron storage cells, including parenchymal cells such as hepatocytes. Removing iron at a faster rate than that excessively reaccumulated by intestinal absorption is critical to negative iron balance, but determining this rate prior to starting depletive treatment is difficult due to individual differences between hemochromatosis patients. It should also be recalled that hepcidin synthesis can be further suppressed by increasing erythropoiesis as a consequence of erythroferrone production. In practice, the depletive treatment includes two successive phases. The first one, called induction phase, aims at totally removing the iron excess present at the time of diagnosis. It usually consists of weekly venesections (≤7.5 mL/kg body weight per venesection). Once excess iron has been removed, the second phase, called maintenance therapy, aims to avoid recurrent iron overload using lifelong venesections, performed every 1–4 months.

### 3.2. Use of Chelation Therapy

Another way to remove iron is the use of iron chelators that promote iron mobilization and excretion. However, in contrast to venesections, these drugs, including mainly today new oral iron-chelators, may have potential side effects [97]. Therefore during genetic hemochromatosis, oral chelation is only used, and as an off-label drug, in rare situations including cardiac failure, recent cerebral ischemic stroke, venous access problems limiting the possibility of venesections, or psychological intolerance to venesections.

### 3.3. Biochemical Follow-Up of Venesection Therapy

Three main parameters are classically followed: plasma ferritin and transferrin saturation levels that reflect the efficacy of iron depletive treatment, whereas hemoglobin levels reflect the tolerance of venesections. During the induction phase ferritinemia—that reliably reflects the amount of iron excess in hemochromatosis (provided other frequent acquired causes of hyperferritinemia have been excluded, such as inflammation, alcoholism, cytolysis or metabolic syndrome; genetic hyperferritinemia, as seen in the ferritin-cataract syndrome, is much rarer)—is the first parameter that decreases. This decrease is only slowly progressive, especially in patients exhibiting a severe iron load phenotype. Conversely, the decrease of transferrin saturation levels, that reflect plasma iron bioavailability, is a very late event. Normalization of transferrin saturation occurs only when the induction treatment is in its final phase. It is noteworthy that during hemochromatosis, despite the impressive frequency of venesections, hemoglobin levels remain stable due to the high capacity of cellular iron release by ferroportin hyperactivity, related to hepcidin deficiency. However, transient hypoxia and relative iron deficiency occurring after venesection, together with increased erythropoiesis, tend to decrease hepcidin plasma levels, thus contributing to further enhance iron entry into the plasma [98,99]. When considering NTBI during the induction therapy, its plasma concentration decreases parallel to transferrin saturation levels [55]. Altogether, these data suggest that induction therapy must be fully completed to remove iron excess, but also to prevent the appearance of toxic forms of iron, including plasma NTBI.

During the maintenance phase, it is essential to pursue the patient follow-up in order to ideally maintain both transferrin saturation and ferritin levels in the normal range [47,95].

## 4. Iron Removal Is not the Unique Therapeutic Target in *HFE*-Related Hemochromatosis

### 4.1. Other Preventive Actions to Avoid Iron Overload Complications

Besides iron removal, it is important to prevent associated causes of organ damage, as mentioned previously. This holds especially true for hepatic lesions knowing the impact of excessive alcohol intake and viral hepatitis as cofactors of liver fibrosis, cirrhosis, and hepatocellular carcinoma. Moreover, NAFLD (Non Alcoholic Fatty Liver Disease) with the risk of NASH (Non-Alcoholic Steato-Hepatitis) represents an additional risk for hemochromatosis patients. The practitioner must complete the iron removal approach by nutritional recommendations on alcohol and glucido-lipidic regimens [47,95]. Moreover, avoiding the occurrence of viral B hepatitis through vaccination is critical. In addition, tea [100] has been demonstrated to limit iron absorption, and therefore, could be used as adjuvant to avoid recurrence of excessive iron stores after completion of iron removal. In the same way, the use of therapeutic oral calcium channel blockers has also been proposed due to its positive impact on urinary iron excretion related to DMT1 activity increase in the kidney [101]. The use of proton-pump inhibitors could also represent an interesting adjunct [102].

### 4.2. The Development of a Pathophysiological Treatment Is a Major Goal

Iron removal is a treatment that only cures iron excess, but clearly does not correct the hepcidin deficiency that generates iron metabolism disturbances, and some clinical observations do suggest that the removal of iron excess is not sufficient to treat hemochromatosis. Considering arthropathy, it is noteworthy that, despite a well-conducted iron depletive treatment, the symptomatology may persist and even become more severe [76]. This suggests that: (i) synovial iron deposition [103] may represent an inaccessible compartment for iron depletion by venesections because synovial fluid is not contiguous with serum; (ii) synovitis, that has been documented in hemochromatosis [104,105] is irreversible despite effective iron depletion; and/or (iii) hepcidin deficiency leading to iron excess could be involved directly in symptomatic arthropathy and/or (iv) the mutations of the *HFE* gene, that encodes an HLA-like class I protein, could be involved directly in disease expression [106].

Considering the biochemical follow-up of patients, it is noteworthy that transferrin saturation levels may be found to be frequently increased during the maintenance therapeutic period, despite the absence of increased body iron stores. This could contribute to the appearance of clinical manifestations such as fatigue and arthropathy. It should be emphasized that, during the maintenance period, serum hepcidin levels are even lower than in iron overloaded patients [98,107]. Such hypohepcidinemia is expected to favor transferrin saturation increase and the appearance of NTBI that could participate in the arthritis of hemochromatosis patients. 

Targeted treatment in the setting of hepcidin deficiency itself could represent an efficient way, as suggested by clinical observations obtained in hemochromatosis patients that have been transplanted for hepatocellular carcinoma [108]. The development of substitutive hepcidin treatment and/or of drugs stimulating hepcidin expression is under development and could be useful to complete the therapeutic arsenal, as suggested by results obtained with mini-hepcidins [109] and BMP known to induce hepcidin expression [110]. Clinical trials with hepcidin supplementation are ongoing (Clinical.trial.gov). Using antisense oligonucleotides for increasing hepcidin synthesis [111] or ferroportin antagonists [112] represents also further interesting innovative approaches. These different treatments could be especially indicated as an adjunct to venesections during the induction phase, and possibly as replacing venesections during maintenance therapy. Considering the overall safety and efficacy of the venesections, every putative treatment of hemochromatosis targeting iron metabolism must be without side-effects and easy to follow, suggesting especially that oral treatments providing exogenous hepcidin or inducing endogenous hepcidin synthesis might be preferable.

## 5. Conclusions

Iron remains the major therapeutic target during *HFE*-related hemochromatosis. However, targeting directly the iron stores by iron depletive treatment does not correct the pathophysiological defect of the disease. The development of treatments aimed at restoring iron homeostasis, especially by correcting hepcidin deficiency, could be useful to optimize the treatment, especially during the maintenance phase by controlling transferrin saturation with hopefully a favorable impact on the arthritis of hemochromatosis patients.
